# Value of measuring waist circumference in primary care: a mixed-methods study

**DOI:** 10.3399/BJGP.2025.0395

**Published:** 2026-06-16

**Authors:** Willemijn J van den Hout, Petra G van Peet, Mattijs E Numans, Sebastiaan C Boone, Lieke Raas, Saskia Le Cessie, Frits R Rosendaal, Dennis O Mook-Kanamori, Renée de Mutsert

**Affiliations:** 1 Department of Public Health and Primary Care, Leiden University Medical Center, Leiden, Netherlands; 2 Department of Clinical Epidemiology, Leiden University Medical Center, Leiden, Netherlands

**Keywords:** waist circumference, cardiometabolic risk factors, obesity, preventive medicine, incidence, abdominal fat, cardiovascular diseases

## Abstract

**Background:**

It remains a challenge for GPs to identify all patients at increased risk of cardiovascular disease (CVD) in their practice, as performing cardiovascular risk assessment in every single patient is not feasible in routine practice. There is need for a simple tool to screen who is eligible for cardiovascular risk assessment and identify those at increased CVD risk.

**Aim:**

To investigate the value of measuring waist circumference in primary care by investigating: current recording practices of GPs; barriers and facilitators; and its contribution as a screening tool in the identification of patients at increased risk of CVD.

**Design and setting:**

A mixed-methods study was conducted in Dutch primary care.

**Method:**

We investigated the following three datasets: routine data from general practices (*n* = 676 708 health records of adults); qualitative data from six focus groups (*n* = 21 GPs); data from the Netherlands Epidemiology of Obesity (NEO) study (*n* = 6671 middle-aged individuals).

**Results:**

Between 2012 and 2023, incidence rates of recorded waist circumference by GPs decreased from 47 to 3 per 1000 person-years. Barriers to GPs measuring waist circumference included discomfort, inability to measure it accurately, lack of measuring tape, and perceived uselessness. Facilitators included knowledge that increased waist circumference is a cardiovascular risk factor. In the NEO study population, after excluding patients already treated for the prevention of CVD (*n* = 2407), 1731 patients were at increased risk of CVD (*n* = 1113 intermediate risk, *n* = 618 high risk). Measuring waist circumference would identify 89% (993/1113) of those at intermediate and 93% (575/618) of those at high predicted cardiovascular risk.

**Conclusion:**

Waist circumference measurement may serve as a valuable screening tool to select patients eligible for cardiovascular risk assessment to identify those at increased risk of CVD in primary care. Since GPs currently rarely measure waist circumference, inclusion in guidelines and addressing identified barriers and facilitators is warranted.

## How this fits in

Cardiovascular risk assessment in all patients in primary care is challenging and often not feasible in routine practice. There is need for a simple tool to screen who is eligible for cardiovascular risk assessment and identify those at high cardiovascular disease (CVD) risk. This study investigated the value of measuring waist circumference in primary care. Waist circumference is rarely recorded by GPs, mainly owing to barriers such as lack of a measuring tape, feeling discomfort, and perceived usefulness. Measuring waist circumference would identify 89% of patients at intermediate and 93% of patients at high predicted cardiovascular risk. Waist circumference measurement may serve as a valuable screening tool to select patients eligible for cardiovascular risk assessment to identify those at increased risk of CVD. Since GPs currently rarely measure waist circumference, inclusion in guidelines and addressing identified barriers and facilitators is warranted.

## Introduction

In the current Dutch guidelines for cardiovascular risk management in primary care,^1^ which is based on European guidelines,^2^ obesity (defined by body mass index [BMI] ≥30 kg/m^2^), but not increased waist circumference, is included in the criteria for the selection of patients eligible for a cardiovascular risk assessment.^1^ Only in individuals who are eligible for cardiovascular risk assessment, a predicted cardiovascular risk is calculated using the Systematic Coronary Risk Evaluation-2 (SCORE2) to estimate the 10-year fatal and non-fatal cardiovascular risk and treatment options are considered.^1,3^ However, individuals with a BMI <30 kg/m^2^, but with an increased waist circumference, are also at increased risk of cardiovascular disease (CVD),^4–7^ and therefore individuals without obesity but with an increased risk of CVD may be overlooked. Whereas it has been previously shown that adding waist circumference to current risk scores does not improve the prediction of CVD^4,5^ in patients who are identified for cardiovascular risk assessment, we hypothesise that it may help to select patients eligible for cardiovascular risk assessment to identify those at increased risk of CVD.

In daily clinical practice, it is not feasible to invite all patients for cardiovascular risk assessment owing to time constraints, the largely demand-driven nature of primary care, and the fact that many patients visit their GP with unrelated complaints. This approach may leave individuals who do not present with cardiovascular-related complaints unassessed, resulting in missed opportunities to identify and treat those at increased cardiovascular risk. Measuring waist circumference could help address this gap and may serve as a tool to select patients who are eligible for cardiovascular risk assessment and may benefit from early treatment. As an easy, low-cost, and non-invasive measure, it is particularly suitable for use during routine consultations.

Body height and weight are frequently measured, and BMI (weight/height squared) is recorded in electronic health records in some countries, while in others it is not.^8–12^ In contrast, waist circumference is rarely assessed in primary care.^13–16^ To address this issue, it is crucial to understand the barriers and facilitators of healthcare providers concerning measuring waist circumference, as this could enhance patient care and health outcomes.^7^ Few studies have explored these barriers and facilitators. Identified barriers in these studies include lack of time and the physician feeling uncomfortable.^13,17^

The aim of this study was therefore to investigate the potential value of measuring waist circumference in primary care. The study focused on three areas. First, the study looked at the current recording practices of GPs regarding waist circumference measurements. Second, it focused on the barriers and facilitators of GPs for measuring waist circumference. Third, it investigated the contribution of measuring waist circumference as a screening tool for the selection of patients in general practice who are eligible for cardiovascular risk assessment to identify those at increased risk of CVD.

## Method

**Figure 1.**
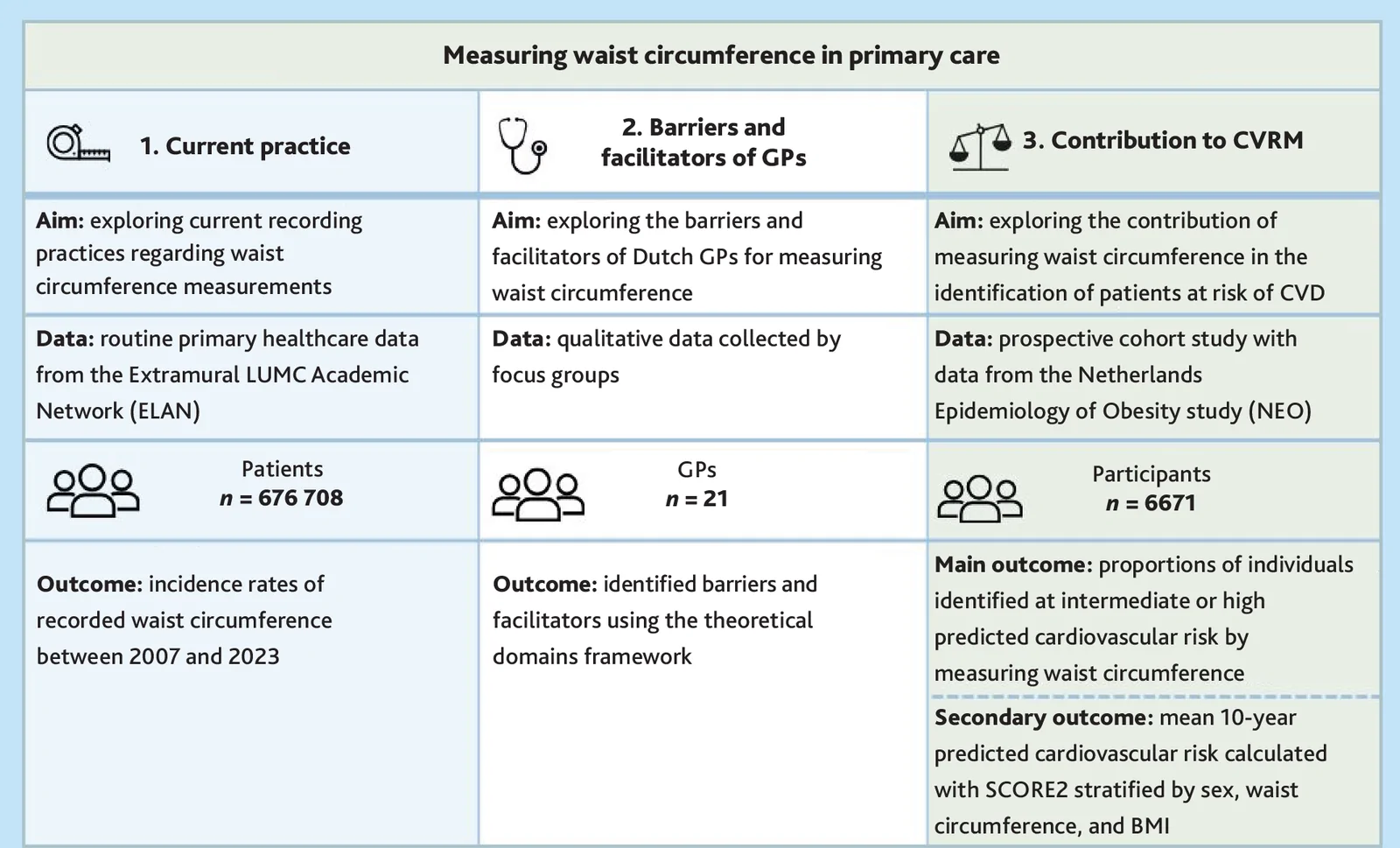
An illustration of the study methods using a mixed-methods approach with three different datasets. BMI = body mass index. CVD = cardiovascular disease. CVRM = cardiovascular risk management. LUMC = Leiden University Medical Center. SCORE2 = Systematic Coronary Risk Evaluation 2.

A mixed-methods approach was used based on data from three different datasets (Figure 1).

### Current recording practices

#### Study design and study population

For our first aim, we used routine collected healthcare data from 676 708 individuals of 152 general practices from the Extramural LUMC (Leiden University Medical Center) Academic Network (ELAN). ELAN is a regional integrative population-based data infrastructure in which medical, social, and public health data are linked at the patient level from the greater The Hague and Leiden area.^18,19^ The study population used for our first aim was also included in a previous study.^12^ However, measuring waist circumference had not been investigated and reported previously. Individuals were included in the analyses when they were registered between 1 January 2007 and 30 June 2023 at a general practice participating in the ELAN database and aged >18 years. The study design and exclusion criteria are described elsewhere.^12,19^

#### Data collection

For waist circumference as outcome, we used the values coded within the structured electronic health records as a laboratory result.

Age, year of birth, and sex were derived at cohort entry. For BMI, we used height, weight, and BMI that was coded within the structured electronic health records as a laboratory result. BMIs were derived from an already available BMI (automatically calculated by the GPs information system), or BMI was calculated using a recorded height and weight on the same date or a recorded weight and a previously recorded height. BMI was calculated by dividing weight in kilograms by the square of height in metres. BMI measurements between 17 kg/m^2^ and 50 kg/m^2^ were included in our analysis. We assessed the data for extreme values and inconsistencies, removing less than 2% of records owing to inadequate or extreme values for height, weight, and BMI. Values <17 kg/m^2^ or >50 kg/m^2^ were excluded, as they may represent measurement or recording errors.

#### Statistical analysis

Baseline characteristics were expressed as median (25th, 75th percentiles) or as percentage. Follow-up time in person-years was calculated from cohort entry (aged ≥18 years and registered in a general practice from 1 January 2007) until deregistration with a participating general practice, death, or end of the study period (30 June 2023).

We estimated the incidence rates of a recorded waist circumference per 1000 person-years for each calendar year from 2007 to 2023. If multiple recorded waist circumferences for an individual were recorded within the same year, only the first recorded waist circumference in that year was included. Individuals were censored after their first recorded waist circumference for that calendar year but were included again in subsequent years. Furthermore, in the electronic health records of the *n* = 676 708 individuals, all BMI recordings between 17 kg/m^2^ and 50 kg/m² were selected. For each of these BMI values, we assessed the frequency of waist circumference measurements taken on the same day. This aimed to determine the frequency of waist circumference recordings for each recorded BMI value within 17–50 kg/m^2^, to identify at which BMI values, waist circumference is most frequently recorded.

### Barriers and facilitators of GPs

#### Study design and study population

For our second aim, we conducted a qualitative focus group study with 21 Dutch GPs to explore the barriers and facilitators to diagnosing obesity in primary care, including the use of a waist circumference measurement.

#### Data collection

The qualitative data used for the second aim were derived from van den Hout *et al.* ^20^ In this previous study, barriers and facilitators to diagnosing obesity were explored. At the start of the focus group sessions, we explained that diagnosing obesity referred to measuring height and weight (for BMI calculation), and preferably also waist circumference, and recording these values in the electronic health records. Participants were reminded of this throughout the discussions. Although waist circumference was not addressed through a dedicated question, participants frequently raised it when discussing the barriers and facilitators to diagnosing obesity. In the present study, we conducted a more in-depth analysis focusing specifically on the barriers and facilitators mentioned by GPs regarding measuring waist circumference. This analysis is presented in greater detail here.

We used purposive sampling to recruit a heterogenous sample of GPs in terms of age, sex, working experience, GP practice setting, and patient populations. Focus groups were organised with three to five GPs, and new groups were added until data saturation was reached, that is, until no new themes were brought forward. Details of the data collection process are described elsewhere.^20^

#### Analysis

Details of the analysis process are described elsewhere.^20^ In short, the transcripts were analysed using a thematic analysis approach using Atlas.ti (version 22). All fourteen theoretical domains of the refined theoretical domains framework (TDF) were used for deductive coding. The TDF is a framework specifically designed to understand determinants of healthcare professional behaviour.^21–23^ Each domain of the TDF relates to a component in the overarching ‘Capability, Opportunity, Motivation, and Behaviour’ (COM-B) model. This model identifies three key factors that need to be present for any behaviour to occur: capability, opportunity, and motivation. Barriers and facilitators using the TDF, and the overarching COM-B model were identified for measuring and recording waist circumference.

### Contribution of measuring waist circumference to cardiovascular risk management

#### Study design and study population

For our third aim, data from the Netherlands Epidemiology of Obesity (NEO) study, a population-based cohort study of 6671 individuals were used. Inclusion criteria for participating in the NEO study were men and women aged between 45 and 65 years with a self-reported BMI of ≥27 kg/m^2^, living in the greater area of Leiden (in the West of the Netherlands). In addition, all inhabitants aged between 45 and 65 years from one municipality (Leiderdorp) were invited via the registry of the municipality. This population has a BMI distribution similar to that of the Dutch general population. Participants were invited to a baseline visit between September 2008 and September 2012 at the NEO study centre of the LUMC. At baseline, participants completed several questionnaires to report demographic and clinical information and underwent anthropometric measurements and blood sampling. The study design and population have been described in detail elsewhere.^24^

For the present analysis, we first determined from the total study population who were already treated by their GP to prevent CVD: those using lipid-lowering or antihypertensive treatment (*n* = 2407). Then, we created the risk assessment population and to be able to calculate SCORE2 in this we excluded those with missing data for the SCORE2 (*n* = 32). These missing data were owing to unsuccessful baseline measurements: blood samples for cholesterol could not be obtained in 23 patients, blood pressure measurements were unsuccessful in seven patients, and smoking status was unknown in two patients. In the risk assessment population (*n* = 4232), we calculated the predicted cardiovascular risk using SCORE2, to identify those at increased risk of CVD.

#### Data collection.

##### a) outcomes

The main outcome was predicted 10-year fatal and non-fatal cardiovascular risk. We categorised individuals into low, intermediate, and high-risk categories either based on a priori high-risk factor or a calculated predicted risk score with SCORE2.^3^ Individuals classified as high risk a priori were those with pre-existing CVD, diabetes mellitus, chronic kidney disease (moderate and severe), severely increased systolic blood pressure, or diagnosed hereditary dyslipidaemia; this was regardless of their SCORE2 result.^1^ The definitions of each of these risk factors are explained in Supplementary Information S1. For others, the predicted cardiovascular risk score was calculated with SCORE2. For individuals with rheumatoid arthritis, risk scores were multiplied by 1.5. The calculated predicted cardiovascular risks were categorised into low, intermediate, and high, based on age and predicted cardiovascular risks, in accordance with the Dutch guidelines for cardiovascular risk management (Supplementary Information S2).^1^

##### b) covariates

The variables used in the SCORE2 were collected at baseline: age, sex, systolic blood pressure, total cholesterol/high-density lipoprotein (HDL) cholesterol ratio, and smoking status. BMI was calculated by dividing the weight by the height squared (kg/m^2^) categorised into normal (<25 kg/m^2^), overweight (25–30 kg/m^2^), and obesity (≥30 kg/m^2^). Waist circumference was measured with a measuring tape placed midway horizontally between the lower costal margin and the iliac crest and categorised into normal (men ≤94 cm, women ≤ 80 cm), increased (men >94–102 cm, women >80–88 cm), and substantially increased (men >102 cm, women >88 cm).^25^

### Statistical analysis

Baseline characteristics of the risk assessment population were expressed as mean (standard deviation; SD), median (25th, 75th percentiles), or percentages. Proportions of patients at low, intermediate, and high predicted risk were calculated within this population to identify individuals at increased risk who had not yet been treated for CVD. Furthermore, proportions of individuals at low, intermediate, and high predicted risk were calculated among patients with an increased waist circumference (men >94 cm, women >80 cm) and compared with the total number of patients at low, intermediate, and high predicted risk in the risk assessment population, to determine what proportion of patients could have been identified through a waist circumference measurement. Finally, mean predicted risk scores with corresponding 95% confidence intervals (CIs) were calculated, stratified by BMI, waist circumference, and sex. Subsequently, patients with an increased waist circumference were selected for further analysis.

## Results

### Current recording practices

**Figure 2.**
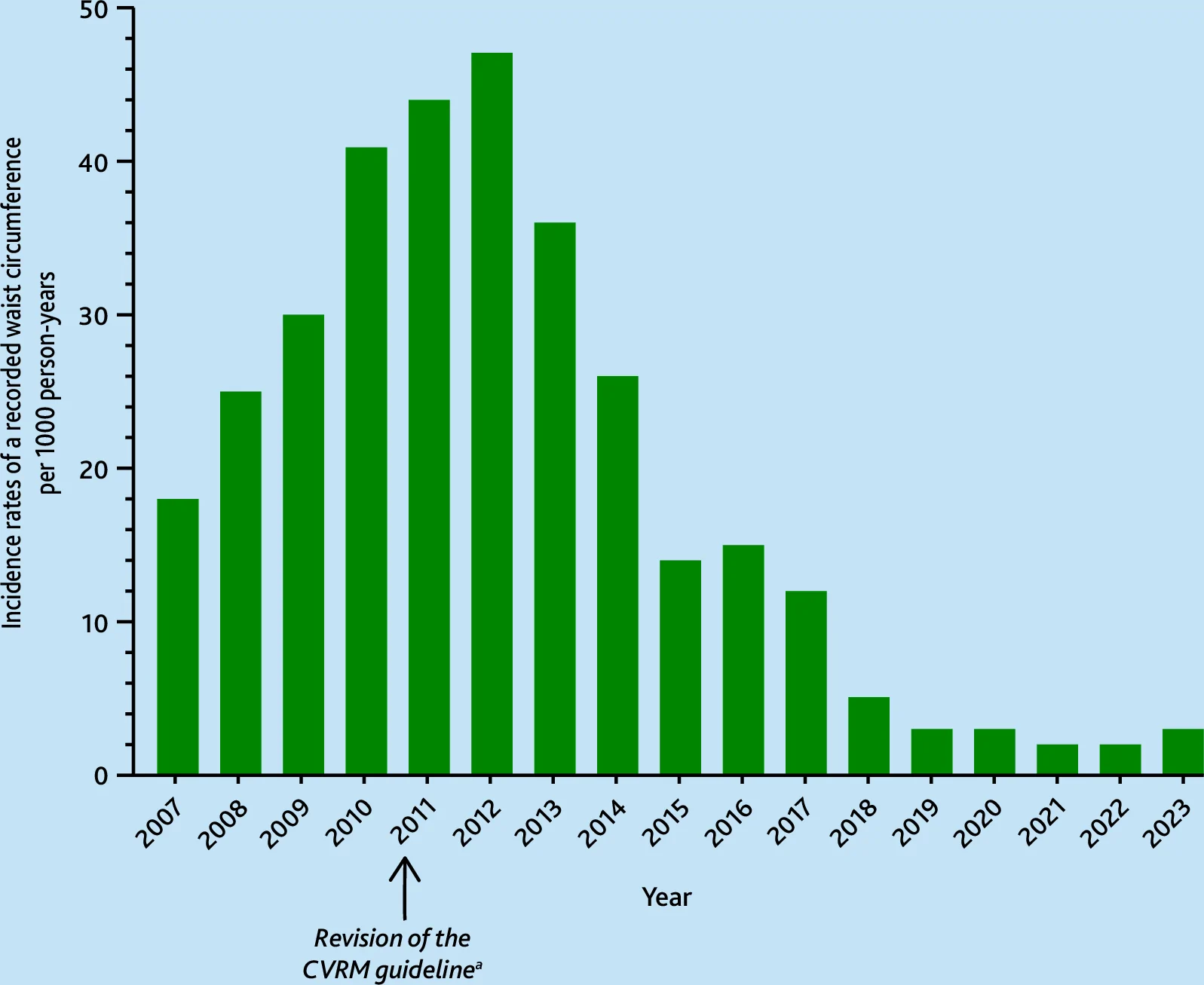
Incidence rates of a recorded waist circumference of *n* = 676 708 electronic health records of Dutch GPs between 2007 and 2023. ^a^The national guideline of the Dutch College of General Practitioners: cardiovascular risk management. CVRM = cardiovascular risk management.

This analysis included 676 708 individuals (Supplementary Table S1) with median of 7.5 person-years (interquartile range 2.8–15.5 person-years) of follow-up in a general practice, and a total of 131 487 waist circumferences recorded. Of the total population, *n* = 45 410 (6.7%) had at least one recorded waist circumference. From 2007 to 2012 the incidence rate of a recorded waist circumference increased with 17 to 47 per 1000 person-years. Between 2012 and 2023, the incidence rate of a recorded waist circumference decreased to 3 per 1000 person-years (Figure 2). For *n* = 128 623 (97.8%) recorded waist circumferences, a BMI was also recorded at the same date. Waist circumference was most often recorded in individuals with BMIs between 25 kg/m^2^ and 33 kg/m^2^ (Supplementary Figure S1).

### Barriers and facilitators of GPs

**Figure 3.**
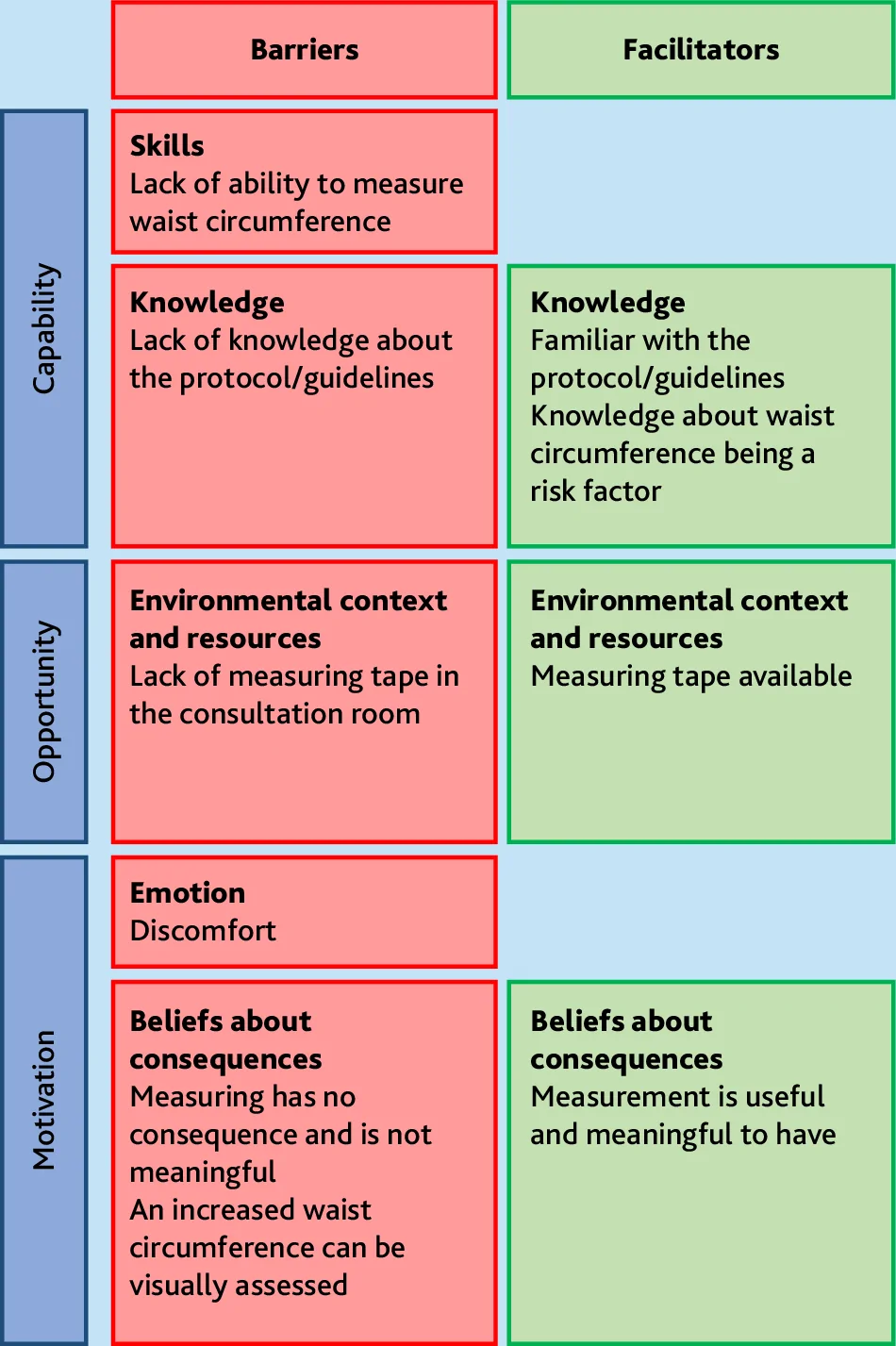
Barriers and facilitators of Dutch GPs in measuring and recording waist circumference.

We reached data saturation after six focus groups with three to five GPs (*n* = 21). The GPs had a mean age of 49 years (range 33–66 years) and the majority were women (76.2%) (Supplementary Table S2). Barriers and facilitators for measuring waist circumference, structured into the three COM-B components with the related domain of the theoretical domains framework in brackets, are described below (see also Figure 3).

#### Capability

For capability, mainly topics belonging to the domain ‘knowledge*’* were mentioned. A facilitator for measuring waist circumference was GPs’ knowledge that an increased waist circumference is a risk factor for developing CVD (knowledge). Additionally, some GPs had familiarity with the guidelines for measuring waist circumference, which served as a facilitator, while other GPs did not, which acted as a barrier (knowledge):

*‘I think it is less common in women, but, um, those men with abdominal obesity, they all get heart attacks.’* (GP2)

As a barrier, some GPs did indicate a lack of skill in measuring waist circumference. On the other hand, as a facilitator, some GPs expressed they were able to measure waist circumference. However, their explanations on how to measure it did not always align with the guidelines on how to measure waist circumference (skills):

*‘Yes, well, I think, um, over the navel, but that probably would not be right.’* (GP1)

*‘... I thought 2 cm below the navel.’* (GP3)

#### Opportunity

As a barrier, GPs mentioned the absence of measuring tape in their consultation room for measuring waist circumference (*environmental context and resources).*

#### Motivation

Some GPs mentioned that measuring waist circumference felt uncomfortable, mainly because of the procedure itself (*emotion*):

*‘... if they* [*patients*] *are really fat, you cannot even get around them.’* (GP8)

*‘... you are kind of in an embrace* [laughter] *they can hold him* [*measuring tape*] *for a moment.’* (GP7)

The most important barrier, however, was related to the domain ‘beliefs about consequences’. Many GPs considered not to measure waist circumference since they felt it had no consequence for further management. They also considered it as an unreliable measurement. Additionally, in the domain ‘beliefs about consequences’, some GPs mentioned that they could visually assess if someone had an increased waist circumference thus deeming the measurement unnecessary:

*‘I don't find it very reliable either … and again, it adds nothing to my policy.’ (GP11)*

*‘… but, of course, you can also tell from the body shape whether someone has abdominal obesity. An apple or a pear shape, I can see that without measuring it, I think.’* (GP8)

### Contribution of measuring waist circumference to cardiovascular risk management

From the total NEO study population (*n* = 6671), 36.1% (*n* = 2407) of individuals were already identified and treated by their GP for cardiovascular disease (CVD) prevention. Baseline characteristics of the remaining individuals who were untreated, referred to as the risk assessment population (*n* = 4232), are presented in Supplementary Table S3.

**Figure 4.**
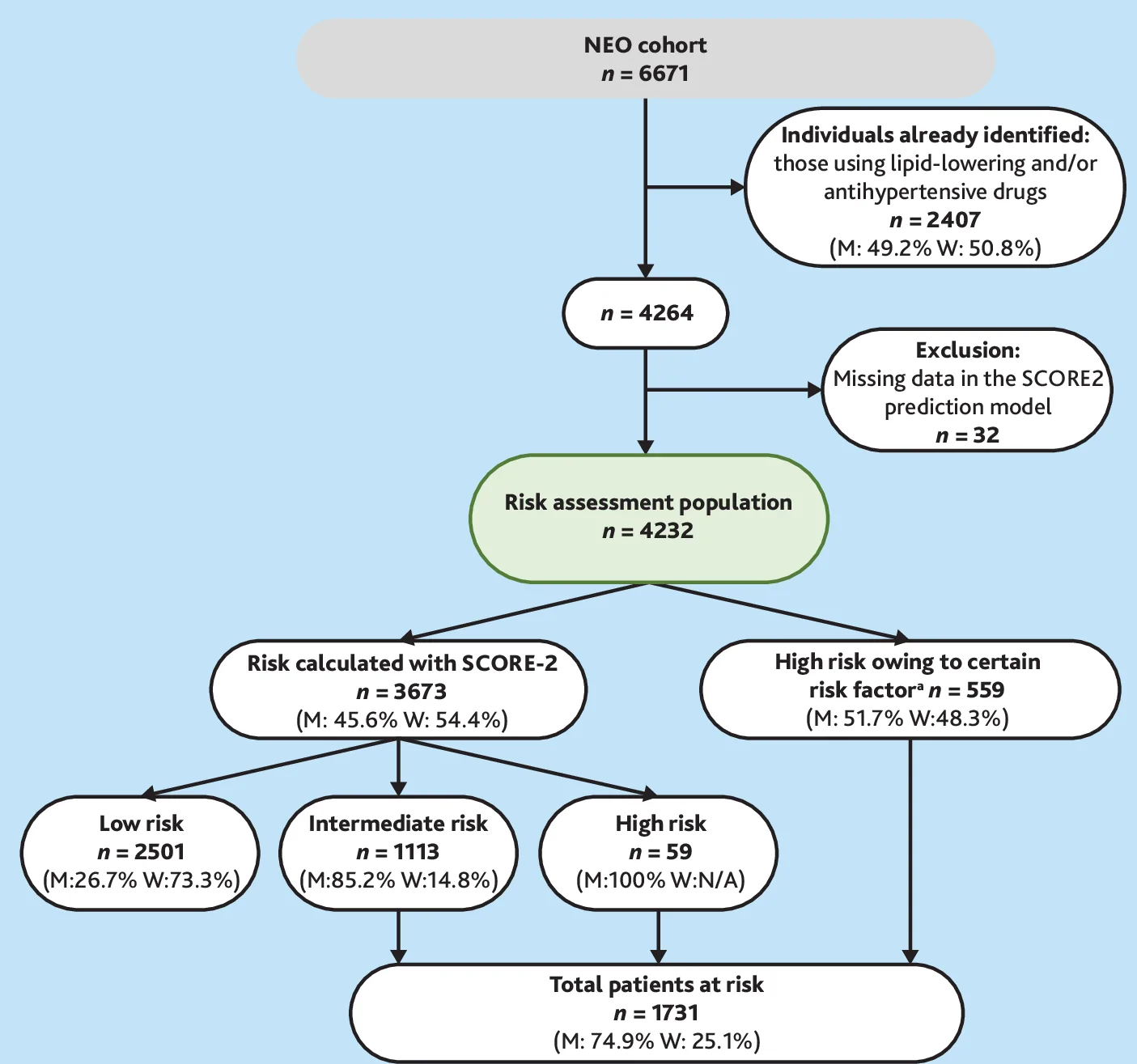
Flowchart of the risk assessment population and the number of individuals at low, intermediate, and high predicted cardiovascular risk in this population.^a^Individuals with pre-existing cardiovascular disease (CVD) *n* = 48, diabetes mellitus *n* = 97, moderate and severe chronic kidney disease *n* = 15, severely elevated blood pressure (systolic >180 mmHg) *n* = 23, diagnosed hereditary dyslipidaemia (low-density lipoprotein [LDL] cholesterol >5 or total cholesterol >8) *n* = 403. SCORE2 = Systematic Coronary Risk Evaluation 2. M = men. W = women.

Within the risk assessment population, 559 individuals were considered a priori at high risk owing to the presence of a specific risk factor. For the remaining 3673 individuals, risk scores were calculated using SCORE2, resulting in 1113 individuals at intermediate predicted risk and 59 at high predicted risk (Figure 4). In total, 1731 individuals (1113 at intermediate and 618 at high predicted risk), representing 25.9% (1731/6671) of the total NEO study population, were not yet identified by their GP but were at increased risk of CVD.

If cardiovascular risk assessment is performed in individuals with an increased waist circumference, 87% of the risk assessment population would be eligible for screening for a cardiovascular risk assessment. Among these, 83.6% of individuals at low predicted risk (2091/2501), 89.2% at intermediate predicted risk (993/1113), and 93.0% at high predicted risk (575/618) would be identified (Table 1). Among those without obesity (BMI <30 kg/m^2^), but with an increased waist, 42.6% (864/2029) patients had an intermediate or high predicted risk and would be identified for further preventive treatment (Table 1).

**Table 1.** Individuals at low, intermediate, and high predicted cardiovascular risk identified by an increased waist circumference^a^, stratified by BMI (risk assessment population of *n* = 4232)

		Low predicted cardiovascular risk (*n* = 2501)	Intermediate predicted cardiovascular risk (*n* = 1113)	High predicted cardiovascular risk (*n* = 618)
Normal waist circumference (*n* = 573)	BMI <25	334	75	27
	BMI 25–30	76	45	15
	BMI ≥30	0	0	1
	Total	**410**	**120**	**43**
Increased waist circumference^a^ (*n* = 3659)	BMI <25	155	23	21
	BMI 25–30	1010	536	284
	BMI ≥30	926	434	270
	Total	**2091**	**993**	**575**
Total		**2501**	**1113**	**618**

^a^An increased waist circumference: men >94 cm, women >80 cm. BMI = body mass index.

**Figure 5.**
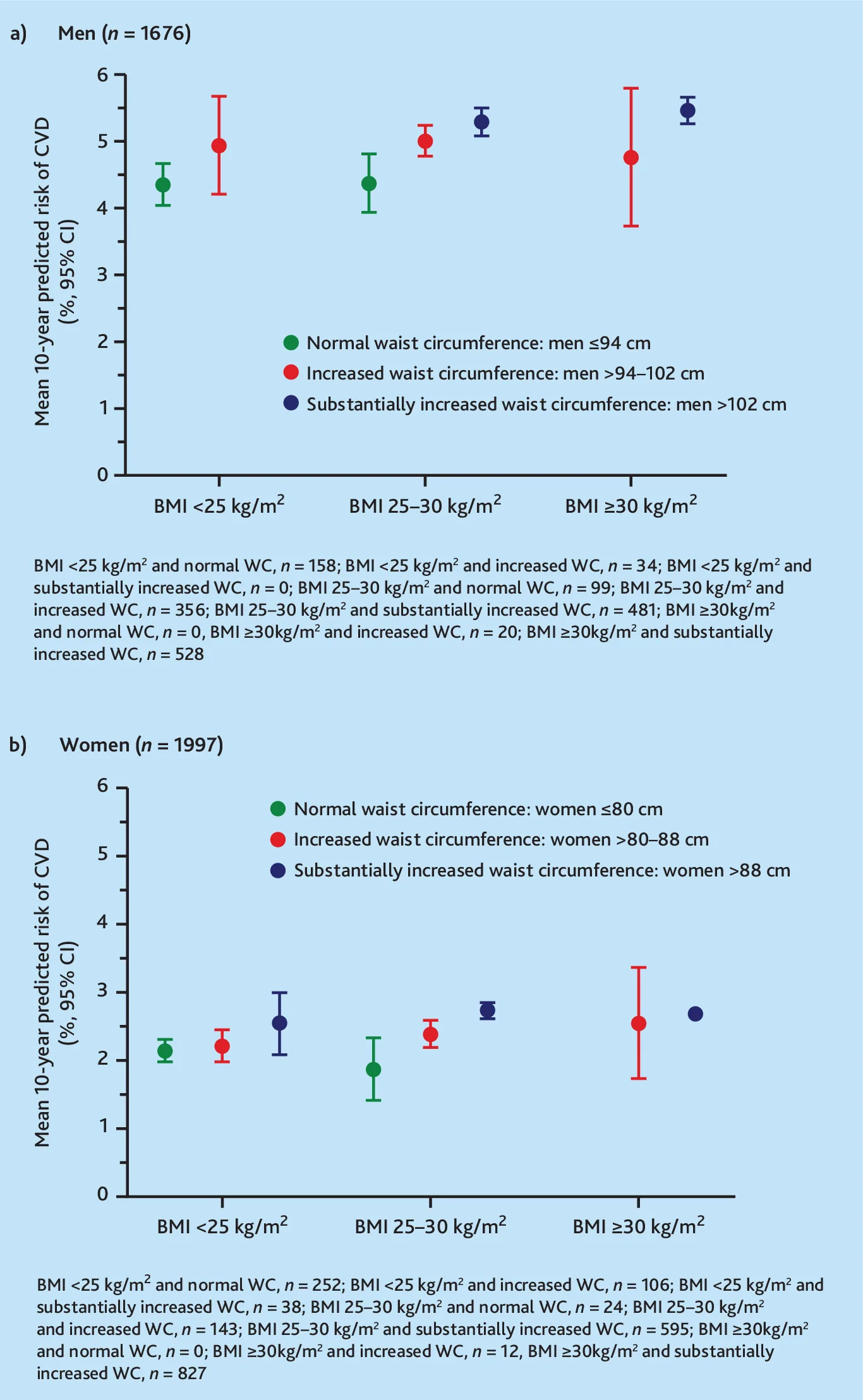
Mean 10-year predicted cardiovascular risk of fatal and non-fatal cardiovascular disease calculated with the SCORE2 stratified by waist circumference, BMI, and sex for a) men (*n* = 1676) and b) women (*n* = 1997). BMI = body mass index. CVD = cardiovascular disease. SCORE2 = Systematic Coronary Risk Evaluation 2. WC = waist circumference.

In the population in which the risk of CVD could be calculated with SCORE2 (*n* = 3673), the mean predicted cardiovascular risk was higher for those with increasing waist circumference in both normal weight and overweight categories (Figure 5). In men with substantially increased waist circumference and overweight, the mean predicted cardiovascular risk was 5.3% (95% CI = 5.1% to 5.5%) compared with 4.4% (95% CI = 4.0% to 4.7%) in those with normal weight and normal waist circumference. In women with overweight and substantially increased waist circumference this was 2.7% (95% CI = 2.6% to 2.9%), compared with 2.1% (95% CI = 2.0% to 2.3%) in women with normal weight and normal waist circumference (Figure 5).

## Discussion

### Summary

In this mixed-methods study, we investigated the value of measuring waist circumference in primary care. Currently, this is not standard practice in primary care in the Netherlands. A waist circumference measurement was recorded in only 6.7% of the GP records of patients aged ≥18 years. Incidence rates of recorded waist circumference decreased from 2012 to 2023 from 47 to 3 per 1000 person-years.

Barriers to GPs measuring waist circumference were feeling discomfort, the inability to measure it accurately, lack of measuring tape, and perceived uselessness. Facilitators included the availability of measuring tape and the comprehension that increased waist circumference is a cardiovascular risk factor. Nevertheless, when waist circumference was measured, this was typically in the range of BMI for patients without obesity but with a large waist and increased CVD risk may be identified.

We showed that measuring waist circumference as a screening tool is a valuable measurement in the identification of patients at increased risk of CVD. Selecting patients with an increased waist circumference for cardiovascular risk assessment identified 89% of those at intermediate and 93% of those at high predicted cardiovascular risk.

Importantly, among patients without obesity, but with an increased waist circumference, 43% had an increased risk of CVD and would not have been identified if only patients with obesity (>30 kg/m^2^) are screened. Given the time constraints and largely demand-driven nature of primary care, it may serve as a less burdensome alternative to assessing multiple risk factors for selecting patients eligible for cardiovascular risk assessment, making risk assessment more targeted and time efficient.

### Strengths and limitations

Strengths of this study are the mixed-methods approach, which ensures that both quantitative and qualitative data were collected. This allows for a broad interpretation of the research results and ultimately, well-tailored interventions that lead to improvements and a more efficient approach in cardiovascular risk management in current practice.

Other strengths of this study are the access to a large population (*n* = 676 708) with routine healthcare data. We also identified barriers and facilitators using a well-established theoretical framework (Theoretical Domains Framework).^21^ Additionally, we had access to extensive and uniform measurements of all information needed for calculating the 10-year cardiovascular risk with SCORE2. Several limitations should be taken into account though. In the routine heathcare data, measured waist circumference could have been missed because analysis was limited within the structured electronic health records, as we did not include free-text data. It is likely that more waist circumferences have been recorded in free text in the electronic health records, as not all GPs translate their medical assessment to accurately coded recordings. This would have led to an underestimation of available recorded waist circumference. It is important to note that routine healthcare data were used. To use these data accurately, we evaluated the data for extreme and non-adequate values and inconsistent records. Only less than 1% of the values were removed, as they may represent measurement or recording errors.

For the qualitative study, focus groups could have yielded socially acceptable answers. Additionally, GPs who attended the focus groups might have had a special interest in obesity and may have been more motivated to optimise the care for patients with obesity. However, only two GPs expressed having a special interest in obesity or lifestyle medicine (Supplementary Table S2).

The first limitation in the NEO study may be the oversampling of individuals with a BMI ≥27 kg/m^2^. This may have led to overestimation of the number of patients with an intermediate or high risk of CVD that can be identified by an increased waist circumference. However, patients who are overweight are more likely to visit their GP for other complaints than patients who are not overweight,^26,27^ and therefore the NEO study population might be viewed as a typical population visiting general practice.

The second limitation in the NEO study is that some individuals in our risk assessment population may already be identified by their GP and monitored accordingly; for example, those with self-reported diabetes, pre-existing CVD, or chronic obstructive pulmonary disease. We included these individuals in our analysis since we were uncertain whether the baseline diagnosis in the NEO study fully aligns with real-life medical records of GPs, leaving it unclear whether these individuals have been identified by their GP. For individuals not yet identified by their GP, measuring waist circumference could be a valuable tool to identify those at increased risk in general practice.

A third limitation of the NEO study is that our study population consists of individuals aged 45–65 years, which limits generalisability to other age groups. However, this age range is appropriate for determining eligibility for cardiovascular risk assessment, as preventive interventions are most effective in this age group. Finally, most participants of the NEO study were White, and because body fat distribution may differ between ethnicities, the results of this study cannot be extrapolated to other ethnic groups.

### Comparison with existing literature

The decrease in recorded waist circumference from 2012 to 2023 may be explained by revisions in the Dutch cardiovascular risk management guidelines. Until 2011, both BMI and waist circumference were recommended as part of the physical examination.^28^ After 2011, the guidelines shifted focus to BMI, with waist circumference listed as an optional additional measurement,^29,30^ possibly owing to its greater intra- and inter-observer variability compared with BMI.^31^ The 2024 updated guidelines reintroduced waist circumference alongside the BMI as part of the physical examination, but waist circumference has not been included as a criterion for cardiovascular risk assessment eligibility.^1^ This might be because it was shown that measuring waist circumference does not improve the prediction of CVD risk beyond established factors such as age, sex, blood pressure, lipids, and smoking behaviour.^4^

However, our results suggest that measuring waist circumference could help as a screening tool to select patients for cardiovascular risk assessment and identify patients at intermediate and high predicted cardiovascular risk, supporting consideration of its role as a screening tool in future guideline updates.

The low measurement rates in our study are consistent with findings from other countries (Canada, the UK, and the US), where waist circumference is also rarely recorded.^13–16^ These trends contradict the increasing awareness that knowledge of waist circumference has added value for risk assessment.^7,32–35^ So, the barriers for measuring waist circumference should be overcome to ease implementation in practice and support guideline adherence.

Previous studies investigated the barriers and facilitators for measuring waist circumference.^13,17^ We confirmed some of the barriers reported by these studies: the discomfort felt by GPs,^13,17^ the perceived usefulness of a measurement,^17^ and the lack of measuring tapes.^13^ In our qualitative study, the most important barriers mentioned were that GPs felt measuring waist circumference had no consequence for further management and that they believed they could visually assess an increased waist circumference. This is particularly important for patients without obesity, in whom an increased waist circumference may be less visible but who are at increased risk of CVD.

Also, considering patient perspectives, Dunkley *et al*, showed that most patients would not feel uncomfortable or embarrassed about having their waist measured. They perceived waist measurement as useful and acceptable, and it could provide an opportunity for open communication with the doctor about health risks and lifestyle changes.^17^

Our results are in line with findings from two other studies that investigated the role of waist circumference in identifying patients at increased risk of CVD,^36,37^ and showed that waist circumference (with cut-offs of 80 cm for women and 94 cm for men) can effectively identify individuals at increased risk, highlighting its value in general practice. Comparing these studies, however, requires careful consideration, as they used different endpoints, such as individual risk factors (for example, hypertension, cholesterol), or other prediction models than the SCORE2 model used in our study, which is currently widely adopted in Europe.

Furthermore, our findings suggest that the population with an increased waist circumference, in addition to the high number of patients identified at high predicted risk, also showed a high observed risk, deeming this population suitable for cardiovascular risk assessment eligibility. The high number of patients identified at high predicted cardiovascular risk by measuring waist circumference can be explained by the strong association between waist circumference and visceral fat, which is associated with CVD.^7,32–35^

Waist circumference is a simple, quick, and non-invasive method to assess abdominal adiposity that is easy to perform in a clinical setting.^7,32,34^ Besides, it is a low-cost, low-risk tool, also useful in settings where other assessments, for example, cholesterol concentrations or blood pressure measurements, are not — or less readily — available.

Even in well-resourced practices, waist circumference can help prioritise patients for further evaluation, reducing the need for multiple separate measurements and supporting a more targeted and time-efficient use of blood tests and blood pressure assessments. It could serve as an opportunistic screening tool (that is, when patients visit their GPs for unrelated reasons) in primary care, thereby facilitating the selection of individuals eligible for cardiovascular risk assessment. Furthermore, waist circumference could also be applied as a screening method in community-based settings, such as pharmacies, gyms, or health fairs, to identify individuals who may need further cardiovascular risk assessment.

We previously showed that selecting patients who are overweight or obese for cardiovascular risk assessment by the GP may help to identify 70% of patients with a treatment indication who were not yet receiving treatment to prevent CVD.^38^ Our present findings may further help to identify patients at increased risk of CVD by measuring waist circumference in those patients who are overweight but with no apparent obesity, enabling further treatment.

Additionally, waist circumference is a measurement that can aid patients to recognise their own condition as it may lead to active attempts and successful weight loss.^39^ Furthermore, it could be used to know which patients should seek and be offered weight management and it enables the effects of weight management and healthy behaviours to be monitored.^7,40^

### Implications for practice

Our findings show that the use of measuring waist circumference as a screening tool may be a valuable addition in general practice. It could be used for the selection of patients eligible for cardiovascular risk assessment to identify those at increased risk of CVD. Since GPs currently do not measure waist circumference frequently, it is not only important to include waist circumference in existing guidelines but also to address the barriers. This can be achieved by providing GPs with training, ensuring access to measuring tapes, and enhancing their understanding of the clinical value of measuring waist circumference, but also in other practice workaround procedures such as creating routine measurement opportunities. Future interventions should focus on tailored interventions to overcome these barriers, ultimately leading to the identification of intermediate and high-risk patients and the prevention of CVD.

## Data

The data underlying this article will be shared on reasonable request to the corresponding author.

## References

[1] Dutch College of General Practitioners (2024). NHG Guideline of the Dutch College of General Practitioners: cardiovascular risk management (M84) (third update) [in Dutch].

[2] Piepoli MF, Hoes AW, Agewall S (2016). 2016 European guidelines on cardiovascular disease prevention in clinical practice: the Sixth Joint Task Force of the European Society of Cardiology and Other Societies on Cardiovascular Disease Prevention in Clinical Practice (constituted by representatives of 10 societies and by invited experts). Developed with the special contribution of the European Association for Cardiovascular Prevention & Rehabilitation (EACPR). Eur Heart J.

[3] SCORE2 Working Group, ESC Cardiovascular Risk Collaboration (2021). SCORE2 risk prediction algorithms: new models to estimate 10-year risk of cardiovascular disease in Europe. Eur Heart J.

[4] Emerging Risk Factors Collaboration, Wormser D, Kaptoge S (2011). Separate and combined associations of body-mass index and abdominal adiposity with cardiovascular disease: collaborative analysis of 58 prospective studies. Lancet.

[5] Pischon T, Boeing H, Hoffmann K (2008). General and abdominal adiposity and risk of death in Europe. N Engl J Med.

[6] Frühbeck G, Busetto L, Dicker D (2019). The ABCD of obesity: an EASO position statement on a diagnostic term with clinical and scientific implications. Obes Facts.

[7] Ross R, Neeland IJ, Yamashita S (2020). Waist circumference as a vital sign in clinical practice: a consensus statement from the IAS and ICCR working group on visceral obesity. Nat Rev Endocrinol.

[8] McLaughlin JC, Hamilton K, Kipping R (2017). Epidemiology of adult overweight recording and management by UK gps: a systematic review. Br J Gen Pract.

[9] Turner LR, Harris MF, Mazza D (2015). Obesity management in general practice: does current practice match guideline recommendations?. Med J Aust.

[10] Baer HJ, Karson AS, Soukup JR (2013). Documentation and diagnosis of overweight and obesity in electronic health records of adult primary care patients. JAMA Intern Med.

[11] Nicholson BD, Aveyard P, Bankhead CR (2019). Determinants and extent of weight recording in UK primary care: an analysis of 5 million adults’ electronic health records from 2000 to 2017. BMC Med.

[12] van den Hout WJ, van Peet PG, Numans ME, Mook-Kanamori DO (2025). Recording practices of body mass index, overweight and obesity by Dutch general practitioners: an observational study. BMC Prim Care.

[13] Gaynor B, Habermann B, Wright R (2018). Waist circumference measurement diffusion in primary care. J Nurse Pract.

[14] Teoh H, Després J-P, Dufour R (2013). Identification and management of patients at elevated cardiometabolic risk in Canadian primary care: how well are we doing?. Can J Cardiol.

[15] Brown I, Stride C, Psarou A (2007). Management of obesity in primary care: nurses’ practices, beliefs and attitudes. J Adv Nurs.

[16] Smith SC, Haslam D (2007). Abdominal obesity, waist circumference and cardio-metabolic risk: awareness among primary care physicians, the general population and patients at risk—the Shape of the Nations survey. Curr Med Res Opin.

[17] Dunkley AJ, Stone MA, Patel N (2009). Waist circumference measurement: knowledge, attitudes and barriers in patients and practitioners in a multi-ethnic population. Fam Pract.

[18] Ardesch FH, Meulendijk MC, Kist JM (2023). The introduction of a data-driven population health management approach in the Netherlands since 2019: the Extramural LUMC Academic Network data infrastructure. Health Policy.

[19] Kist JM, Vos HMM, Vos RC (2024). Data resource profile: Extramural Leiden University Medical Center Academic Network (ELAN). Int J Epidemiol.

[20] van den Hout WJ, Adriaanse MA, Den Beer Poortugael LM (2024). Dutch gps’ perspectives on addressing obesity: a qualitative study. BJGP Open.

[21] Atkins L, Francis J, Islam R (2017). A guide to using the theoretical domains framework of behaviour change to investigate implementation problems. Implement Sci.

[22] Michie S, Atkins L, West R (2014). The behaviour change wheel: a guide to designing interventions.

[23] Cane J, O’Connor D, Michie S (2012). Validation of the theoretical domains framework for use in behaviour change and implementation research. Implement Sci.

[24] de Mutsert R, den Heijer M, Rabelink TJ (2013). The Netherlands Epidemiology of Obesity (NEO) study: study design and data collection. Eur J Epidemiol.

[25] World Health Organization (WHO) (2008). Waist circumference and waist–hip ratio: report of a WHO expert consultation.

[26] Twells LK, Bridger T, Knight JC (2012). Obesity predicts primary health care visits: a cohort study. Popul Health Manag.

[27] Frost GS, Lyons GF, Counterweight Project Team (2005). Obesity impacts on general practice appointments. Obes Res.

[28] Dutch College of General Practitioners (2006). NHG guideline of the Dutch College of General Practitioners: cardiovascular risk management.

[29] Dutch College of General Practitioners (2018). NHG Guideline of the Dutch College of General Practitioners: cardiovascular risk management (M84) (second update) [in Dutch].

[30] Dutch College of General Practitioners (2011). NHG guideline of the Dutch College of General Practitioners: cardiovascular risk management (M84) (first update).

[31] Sebo P, Beer-Borst S, Haller DM, Bovier PA (2008). Reliability of doctors’ anthropometric measurements to detect obesity. Prev Med.

[32] Neeland IJ, Ross R, Després J-P (2019). Visceral and ectopic fat, atherosclerosis, and cardiometabolic disease: a position statement. Lancet Diabetes Endocrinol.

[33] Després JP (2012). Body fat distribution and risk of cardiovascular disease: an update. Circulation.

[34] Pouliot MC, Després JP, Lemieux S (1994). Waist circumference and abdominal sagittal diameter: best simple anthropometric indexes of abdominal visceral adipose tissue accumulation and related cardiovascular risk in men and women. Am J Cardiol.

[35] Boone SC, van Smeden M, Rosendaal FR (2022). Evaluation of the value of waist circumference and metabolomics in the estimation of visceral adipose tissue. Am J Epidemiol.

[36] Han TS, van Leer EM, Seidell JC, Lean ME (1996). Waist circumference as a screening tool for cardiovascular risk factors: evaluation of receiver operating characteristics (ROC). Obes Res.

[37] Siren R, Eriksson JG, Vanhanen H (2012). Waist circumference a good indicator of future risk for type 2 diabetes and cardiovascular disease. BMC Public Health.

[38] de Boer AW, de Mutsert R, den Heijer M (2015). Overweight can be used as a tool to guide case-finding for cardiovascular risk assessment. Fam Pract.

[39] Singh S, Somers VK, Clark MM (2010). Physician diagnosis of overweight status predicts attempted and successful weight loss in patients with cardiovascular disease and central obesity. Am Heart J.

[40] Lean ME, Han TS, Morrison CE (1995). Waist circumference as a measure for indicating need for weight management. BMJ.

